# Europium(III)-Doped Gadolinium(III) Complex for High-Sensitivity Temperature Sensing in the Physiological Range

**DOI:** 10.3390/ma15217501

**Published:** 2022-10-26

**Authors:** Kevin Soler-Carracedo, María Díaz-González, Inocencio R. Martin, Susana Rios, Beatriz Gil-Hernández, Gabriela Brito-Santos, Joaquín Sanchiz

**Affiliations:** 1 Departamento de Física, Universidad de La Laguna, Apdo. 456, E-38200 San Cristóbal de La Laguna, Spain; 2 Instituto Universitario de Materiales y Nanotecnología (IMN), Universidad de La Laguna, Apdo. 456, E-38200 San Cristóbal de La Laguna, Spain; 3 Departamento de Química, Facultad de Ciencias, Universidad de La Laguna, Apdo. 456, E-38200 San Cristóbal de La Laguna, Spain

**Keywords:** hybrid organic-inorganic complex, optical temperature sensor, luminescence, downshifting

## Abstract

A new Eu^3+^-doped Gd^3+^ complex of formula [Eu_0.0135_Gd_0.9865_(pta)_3_me-phen] was synthesized and structurally characterized (Hpta = benzoyltrifluoroacetone, me-phen = 5-methyl-1,10-phenanthroline). The photoluminescence study revealed that when the compound was excited at RT, under a 457 nm continuous laser, the material exhibited high luminescence due to the antenna effect of the ligands, as well as a good balance between the phosphorescence from the spin-forbidden triplet (from the organic ligands), and the characteristic lanthanide f-f transitions. The ratio between the previous emissions drastically changed when the sample was heated up to 62 °C inside a tubular furnace. This ratio was investigated using the luminescence intensity ratio method, to analyze the capabilities of the sample as a temperature sensor. The relative sensitivity reached a maximum of 11.4 °C^−1^ %, maintaining a detection limit below 0.15 °C for the whole temperature range.

## 1. Introduction

In recent years, much effort has been devoted to developing lanthanide ratiometric thermometers based on the temperature-induced changes in the luminescence intensity of two different transitions [1,2]. Lanthanide ions are fundamental in all luminescence science research and are usually employed in optical sensors due to their interesting optical properties. In particular, numerous research works have investigated trivalent lanthanide ions, so their optical properties are relatively well known [3]. These ions feature 4f-electrons’ shielding, which is related to their characteristic sharp absorption and emission spectra covering a wide UV–Vis–NIR range.

The host material plays an essential role in the design of optical devices, as its vibratory properties can influence the optical behavior of the dopant ion. Many of the transition lines (of practical importance) of lanthanide ions originate from excited levels with a small energy gap. Therefore, material hosts with lower phonon energy are necessary, in order for the radiative transitions of active ions to dominate over non-radiative losses. Furthermore, lanthanide ions present weak light absorption, which translates into weak luminescence, since the luminescence intensity is proportional to the absorption [4,5]. Hybrid organic–inorganic compounds can overcome this problem by providing an intense absorption band from an organic ligand that will transfer the higher absorbed-light energy to the lanthanide ion. This effect is known as the antenna effect (or sensitization) [6]. On top of that, they are easy to process (and, therefore, to mix), to create composites that can combine temperature sensing with other novel and interesting applications [7,8,9].

In this work, we study the capability of the Ln(III) complex [Eu_0.0135_Gd_0.9865_(pta)_3_me-phen] as an optical temperature ratiometric sensor. Ratiometric thermometers based on dual-emission provide a self-calibrated temperature readout that is unaffected by sensor concentration and/or excitation-signal fluctuations. Thus, these thermometers are more reliable and accurate than thermometers based on the emission intensity of a single transition [10,11,12]. This type of dual-emission measurement is commonly comprehended as being part of the luminescence intensity ratio (LIR) or fluorescence intensity ratio (FIR) techniques, and is based mainly in the exploit of pairs of thermalized energy levels in trivalent lanthanide-doped materials that can be fitted to a Boltzmann equation. This classic approach, has limited sensitivity in the sensors, proportional to the energy gap between the thermalized levels [13]. By using a complex material, combining organic and inorganic emissions, however, it is possible to overcome the previous sensitivity limit of the Boltzmann thermometer. This work may be considered as part of an exciting new trend, related to overcoming the previously-mentioned limit. Other approaches include the combination of linear and non-linear optics, whether by second-harmonic generation [14,15,16] or multilevel cascade [17], as well as the combination of a single-emission band and the conventional Boltzmann ratio [18].

## 2. Experimental Setup

### 2.1. Synthesis

Reagents: all chemicals and reagents were commercially available and used without further purification; specifically, benzoyltrifluoroacetone 99% (Hpta), 5-methyl-1,10-phenanthroline (me-phen) 99%, triethylamine 99%, ethanol, and Eu(NO_3_)_3_·5H_2_O (99.9%), Gd(NO_3_)_3_·6H_2_O (99.9%). All the reactions were performed under a dinitrogen atmosphere.

Synthesis of [Eu_0.0135_Gd_0.9865_(pta)_3_me-phen]: Hpta (163.8 mg, 0.75 mmol) and triethylamine (150 μL, 1.00 mmol) were dissolved in 9 mL of ethanol in a round-bottomed flask. Then, 0.25 mmol of me-phen (48.6 mg) dissolved in 3 mL of ethanol was added. Subsequently, 0.0034 mmol of Eu(NO_3_)_3_·5H_2_O (1.5 mg) and 0.2466 mmol of Gd(NO_3_)_3_·6H_2_O (111.3 mg), for a sum of 0.25 mmol of Ln(NO_3_)_3_, were dissolved together in 3 mL of ethanol and added to the reaction flask. The solution was heated under stirring at 60 °C for 150 min. After that, the solution was cooled to room temperature and 10 mL of deionized water was added. A white precipitate appeared immediately, and the solution was stirred for another 15 min. The product was collected by filtration, washed with 10 mL of water and 5 mL of ice-cooled ethanol, and dried at 60 °C overnight. The crude product was recrystallized by diffusion of n-heptane in an acetonitrile solution of the complex. During the recrystallization process, some crystals were collected for single-crystal X-ray diffraction, to solve the structure, and the rest was ground, for: X-ray powder diffraction, elemental analysis, thermogravimetry and photoluminescence measurements. Synthesised [Eu_0.0135_Gd_0.9865_(pta)_3_me-phen] yield: 210.1 mg (85%). Elemental analysis (%) calculated for C_43_H_28_N_2_Eu_0.0135_Gd_0.9865_O_6_F_9_ (996.85): C, 51.75; H, 2.80; N, 2.81. Found: C, 51.8; H, 2.8; N, 2.9. IR (KBr, ν/cm^−1^): 3070(m), 2921(m), 1612(s), 1577(s), 1527(m), 1319(s), 1292(s), 1187(s), 1139(s), 1078(w), 767(m), 703(m), 603(m), 582(m). UV-vis (ethanol, λ_max_/nm): 232, 266, 324.

### 2.2. General Characterization Methods

FT-IR as KBr disks, in the 400 cm**^−^**^1^ to 4000 cm**^−^**^1^ range, was recorded on a Thermo NicolletAvatar 360 FT-IR spectrometer (Nicolet Instruments, Madison, WI, USA). UV-visible spectra, between 220 nm and 800 nm, with samples dissolved in ethanol, were recorded on a Varian Cary 50 bio UV-Visible spectrophotometer (Agilent Technologies, Santa Clara, CA, USA). X-ray powder diffraction patterns were recorded on a PANanalytical X’pert X-ray diffractometer (Malvern Panalytical, Malvern, United Kingdom) with Cu Kα radiation, 1.54184 Å, at room temperature.

### 2.3. Single-Crystal X-ray Crystallography

A suitable single crystal was selected under a polarizing microscope, taken directly from the mother liquors, and covered with a protective oil before putting it on a 0.05 mm loop. Single-crystal XRD data were collected with an Agilent SuperNova diffractometer (Agilent Technologies, Santa Clara, CA, USA), with a micro-focus X-ray, under Cu-K_α_ radiation (λ = 1.5418 Å). CrysalisPro software (v1.171.41.122a ,Rigaku Corporation, Tokyo, Japan, 2021) was used to collect, index, scale and apply analytical absorption correction, based on the multi-scan method.

### 2.4. Structure Analysis and Refinement

The structure was solved by direct methods, using SHELXT2018/2 [19], and refinement was undertaken via full-matrix least-squares on F^2^, using SHELXL2018/3 [19]. Crystal data and details of the structure refinement are given in Table 1. Crystallographic data for the structure have been deposited in the Cambridge Crystallographic Database with deposition number 2162029. The structure was solved considering all the Ln atoms as Gd, since it corresponds to 98.65 % of the total Ln. The formula and the distances and angles in Table 1 and Table 2 refer to this element.

Aromatic hydrogen atoms and hydrogen atoms in the alpha-position of benzoyltrifluoroacetone ligand’s diketonate group were situated geometrically (C-H = 0.95 Å) and refined using a riding model (AFIX 43) with U_iso_(H) = 1.2 U_eq_(C). The Methyl group’s hydrogens in the phenanthroline ring were also positioned geometrically (C-H = 0.98 angstroms) and refined using a riding model (AFIX 137) with U_iso_(H) = 1.5 U_eq_ (C). This methyl group is disordered over positions 5 and 6 of the phenanthroline ring and was refined with PART instructions, with occupancies of 0.42:0.58. The phenyl ring of the benzoyltrifluoroacetone ligand labelled A was refined using a riding model AFIX 66.

### 2.5. X-ray Powder Diffractograms

**Figure 1:**
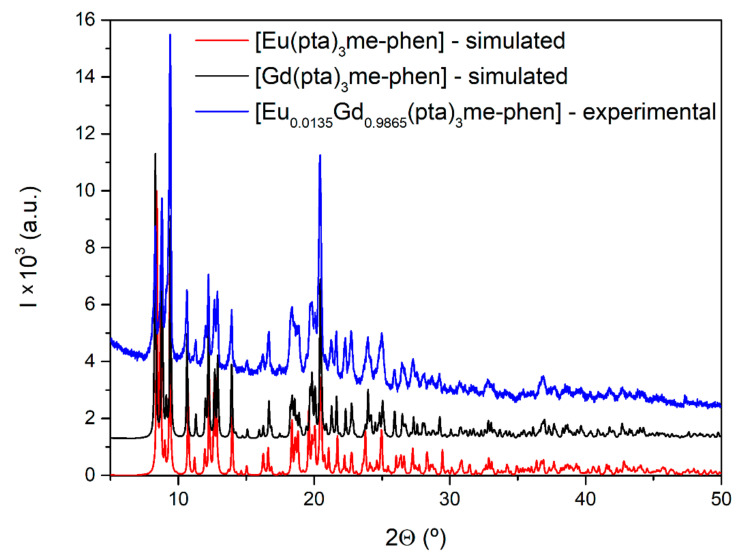
Simulated and experimental powder diffractograms.

There was a perfect match between the simulated (from single-crystal structure) diffractogram and the experimental powder diffractogram (Figure 1), which confirmed the integrity of the product sample and allowed us to use the recrystallized material for the rest of the measurements. Figure 1 also shows the isostructural character of [Gd(pta)_3_me-phen] and [Eu(pta)_3_me-phen] [20].

### 2.6. Optical Characterization

The emission spectra for the sample were obtained by excitation with a 457 nm continuous laser, with the emission focused onto an optical fiber, coupled to a 0.303 m grating single spectrometer (Andor Shamrock SR-303i-A from Andor Technology Ltd, Belfast, United Kingdom). For the detection, a cooled Newton CCD camera (Andor Technology Ltd, Belfast, United Kingdom) was used. All spectra were corrected from the respective spectral response of the equipment.

**Figure 2:**
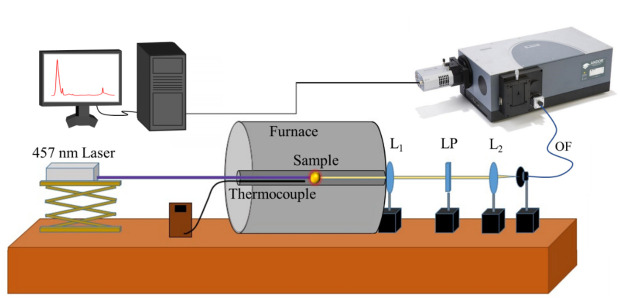
Experimental setup used for the temperature calibration of the emission spectra.

For the temperature calibration, the same setup was used, with the sample placed inside a closed tubular furnace (Gero RES-E 230/3 from Carbolite Gero, Derbyshire, UK), controlled via contact with a type K thermocouple (Figure 2):

where L1 and L2 are lenses, LP is a Long Pass filter and OF corresponds to the optical fiber that collects the signal.

## 3. Results and Discussion

### 3.1. Structure of the Compound

**Figure 3:**
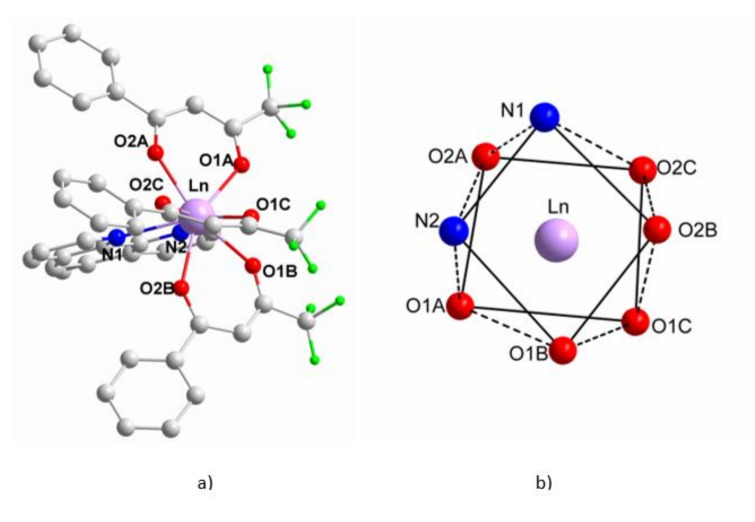
(**a**) molecular structure of [Eu_0.0135_Gd_0.9865_(pta)_3_me-phen], hydrogen atoms omitted for clarity**;** (**b**) square–antiprismatic environment around the Ln^3+^ ion.

[Eu_0.0135_Gd_0.9865_(pta)_3_me-phen] crystallizes in the monoclinic P2_1_/c space-group and has a molecular structure. The Gd^3+^ and the Eu^3+^ ions are randomly dispersed throughout the material and occupy the same crystallographic positions, replacing each other. The Ln^3+^ ions are bound to three β-diketonate (A, B and C) and to one 5-methyl-phenanthroline ligand, (Figure 3). The me-phen ligand shows its typical coordination mode, with the two donor nitrogen atoms directly bound to the Ln^3+^ ion. The pta^-^ ligands bind the Ln^3+^ ion through the two oxygen atoms, forming five-membered chelate rings. The Ln^3+^ atoms are in an eight-coordination structure, with a distorted square–antiprismatic geometry, surrounded by six oxygen atoms from the three diketonate ligands, with distances in the range 2.3361(1) Å to 2.3574(1) Å and, furthermore, surrounded by the two nitrogen atoms of the 5-methyl-phenanthroline, at distances 2.5491(1) Å-2.5880(1) Å, (Figure 3b). The distances and angles are in the expected range, in agreement with similar complexes (Table 2) [21,22].

Table 3 shows the intramolecular hydrogen bonding distances. Those between one of the diketonate oxygen atoms and the closest phenylene hydrogen range from 2.4159(1) Å to 2.4937(1) Å. Additionally, in those between the diketonate hydrogen and one of the F atoms, the range is 2.3378(1) Å – 2.2769(1) Å. These hydrogen bonds block the twist of the phenylene ring with respect to the diketonate group (twisting angles in the range of 13.58° to 24.36°), enhancing the conjugation and the rigidity of the molecule favoring the luminescence process [23,24,25].

**Figure 4:**
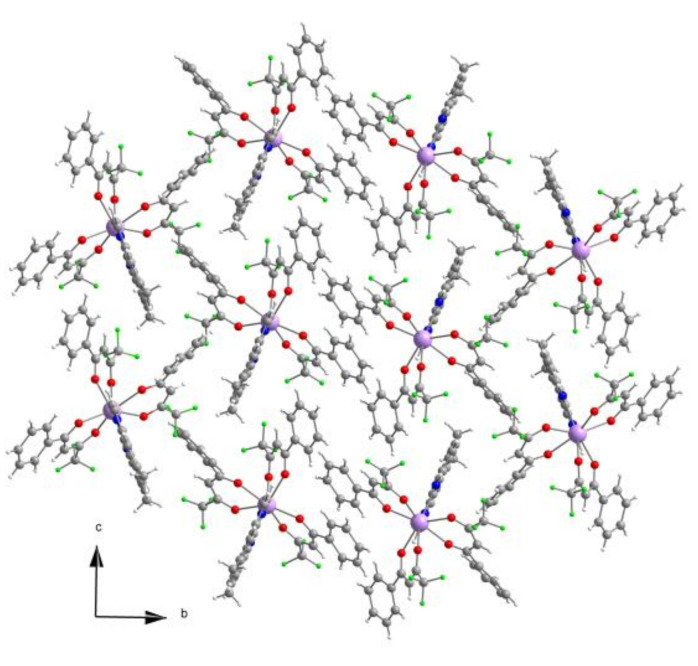
Packing diagram of the structure of [Eu_0.0135_Gd_0.9865_(pta)_3_me-phen].

The packing diagram, Figure 4, shows the molecular structure of the complex. Weak intermolecular interactions favor the complex’s solubility in solvents such as CH_2_Cl_2_ or CH_3_CN. The voluminous character of the ligands and the high coordination number of the complex prevents the entry of water molecules into the coordination sphere of the metal ion, which would deactivate the molecule by non-radiative vibrating processes [25].

### 3.2. Emission Spectrum and Temperature Sensor

**Figure 5:**
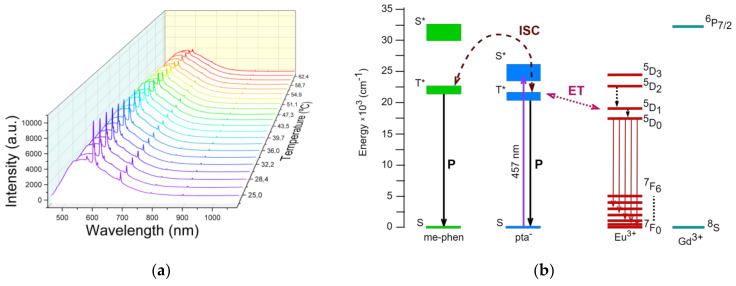
(**a**) Emission spectra of [Eu_0.0135_Gd_0.9865_(pta)_3_me-phen] under 457 nm in the range of 23–62 °C; (**b**) Partial energy-level diagram indicating the transitions observed under the excitation of the 457 nm laser where ISC (intersystem crossing) occurs; ET is energy transfer; P is phosphorescence. Non-radiative relaxation processes are represented by dashed arrows [29].

The emission spectrum of the Eu^3+^-doped Gd^3+^ complex under 457 nm continuous laser excitation is shown in Figure 5a. Two different emissions can be seen superimposed in the spectrum. First, the characteristic emission bands of Eu^3+^ at 595 nm (^5^D_0_→^7^F_1_), 611 nm (^5^D_0_→^7^F_2_), 647 nm (^5^D_0_→^7^F_3_), and 696 nm (^5^D_0_→^7^F_4_); second, a broad emission band centered at 550 nm and related to the T_1_→S_0_ phosphorescence from the organic ligands [26].

According to the spectrum shown in Figure 5a, Figure 5b shows a schematic diagram of the energy levels in the lanthanide ions and the ligands, along with the energy transfers involved in the luminescence process. The 457 nm laser radiation excites the pta^-^ ligand from the ground singlet state to the excited singlet state. At this point, ISC to the excited triplet states of the pta^-^ and me-phen takes place, then energy transfer (ET) to the excited states of the Eu^3+^ ion occurs. Finally, the decay to the ground states of the Eu^3+^ ion produces the luminescence. The laser at 457 nm can also directly excite the Eu^3+^ ion to its ^5^D_2_ state, and after a non-radiative decay to the ^5^D_0_ state, luminescence occurs. These processes compete with the phosphorescence (P) of the ligands. A time decay of 0.44 ms for the Eu^3+ 5^D_0_ state was obtained, which is in good agreement with the literature and can be related to a good sensitization [27,28]. Changes in the temperature produce differences in the relationship between both decay pathways, and this forms the base of temperature sensing. Given the absence of UV-excitation sources, no contribution to the luminescence is expected from the Gd^3+^ ions.

**Figure 6:**
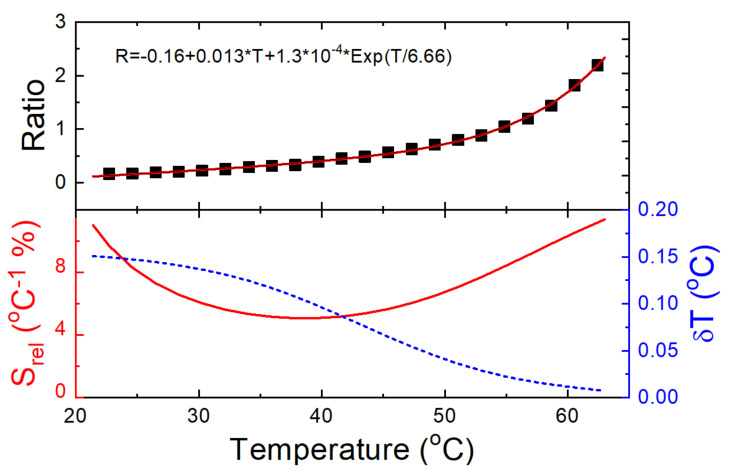
Ratio measurements as a function of temperature (top), relative sensitivity (bottom—continuous red line) and temperature uncertainty (bottom—dashed blue line).

The sample’s response calibration with temperature was performed by introducing the sample into a furnace and heating the system from RT up to 62 °C (Figure 5a). A thermal redistribution of the population was observed and the ratio of intensities between the emission bands of Eu^3+^ and the organic ligands drastically changed, reaching a change of over one order of magnitude for a 40 °C range increase. Following this behavior, the LIR was used to calibrate the temperature between the emission related to the organic compound and the Eu^3+ 5^D_0_→^7^F_2_ (611 nm) emission band (Figure 6). Given the not-well-resolved state of the emissions, the ratio was obtained by means of the separation of chosen wavelength regions. This method was chosen for its simplicity, although it is worth mentioning that spectral deconvolution could lead to a slight increase in terms of relative sensitivity [13]. Integration from 470 to 580 nm was collected for the organic emission, whereas for the Eu3+ transition the integration was collected from 605 to 625, after applying a baseline subtraction. The obtained data were fitted to a Boltzmann-type equation.

To characterize the performance of the sample as a temperature sensor, the relative sensitivity and the temperature uncertainty were obtained and presented in Figure 6. The relative sensitivity is a magnitude defined by the rate at which the measured parameter (Δ) changes with respect to temperature and is given by

(1) $$S_{rel}=\frac{1}{\Delta }\left|\frac{d\Delta }{dT}\right|\cdot 100$$

In this experiment the measured parameter (Δ) was calculated as the ratio between the emission related to the organic compound and the Eu^3+ 5^D_0_→^7^F_2_ (611 nm) emission band (LIR).

In contrast to the absolute sensitivity, the relative sensitivity magnitude allows for the comparison of sensors independently, on the physical parameter analyzed as a function of temperature. Figure 6 shows maximums of 11 °C^−1^ % and 11.4 °C^−1^ % for temperatures of 23 °C and 62 °C, respectively, with a minimum of 5.1 °C^−1^ % at 40 °C. Higher values for the relative sensitivity could be achieved over 62 °C, but they were considered outside the reliability criterion of the intensity of the sensor when above 5% of the noise level [30]. In Table 4, a list of optical temperature sensors based on Eu^3+^-doped lanthanide ions is ranked by relative sensitivity. Outside of this study (as far as can be ascertained), no sensor within this reliability criterion has achieved this high level of relative sensitivity in the physiological range. A sensor by X. Lui et al. [31], with relative sensitivity of 31 %K^−1^ at 4 K, presented values below 1 %K^−1^ in the physiological range. The sensor by X. Yang et al. [32], with a relative sensitivity of 16 %K^−1^ at 383 K, also showed a maximum of 9.01 %K^−1^ over the physiological range, while the sensor by A. Kovalenko et al. [33] was analyzed for a range of temperatures well below this range.

**Figure 7:**
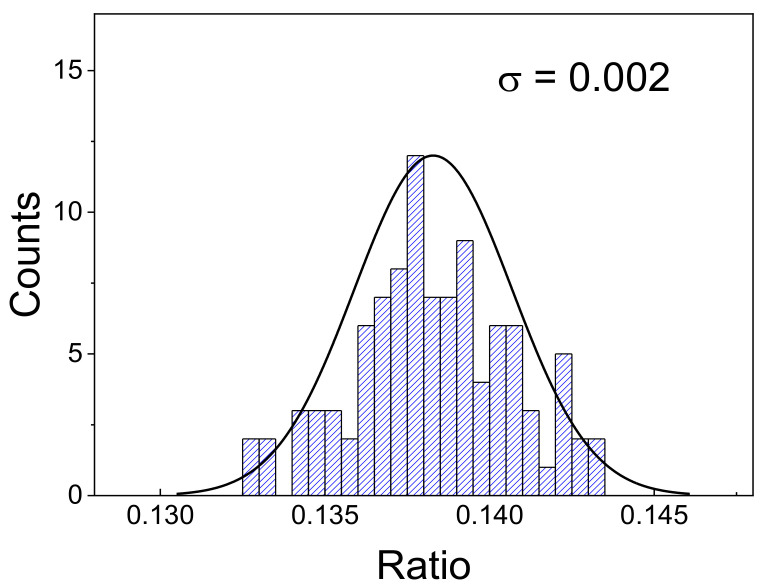
Distribution of the measured parameter (LIR) and the corresponding standard deviation.

Finally, to characterize the error of the sensor, the temperature uncertainty (or temperature resolution) was calculated. Temperature uncertainty refers to the minimum temperature change that can be detected in a given measurement by the sensor and is given by [30]

(2) $$\delta T=\frac{1}{S_{rel}}\frac{\delta \Delta }{\Delta }$$

where δΔ corresponds to the uncertainty in the determination of the Δ. To obtain this last parameter experimentally, 100 measurements were carried out on with the sample at RT, in the same conditions, where the temperature-dependent measurements were undertaken. The resulting LIR readouts and the respective standard deviation are presented in Figure 7. The experimental uncertainty in the determination is considered as the standard deviation for the LIR readouts (δΔ equal to 0.002). Using this parameter in Equation (2), the resulting minimum and maximum temperature uncertainties are 0.008 and 0.15 °C for the temperatures of 62 and 23 °C, respectively (Figure 6). Both results are well below the inner limit of precision that can be found in cell-temperature sensing, among other applications [54].

## 4. Conclusions

A coordination compound combining pta^-^ and me-phen ligands with Eu^3+^ and Gd^3+^ lanthanide ions was successfully synthesized and characterized. When excited under a 457 nm continuous laser, the emission spectrum showed a balanced equilibrium between the broad phosphorescence from the organic ligands and the sharp peaks from the Eu^3+^ at RT. When the sample was heated in the physiological temperature range, up to 62 °C, a drastic change was observed in the intensities between the organic phosphorescence and the lanthanide luminescence. The ratio of these intensities was analyzed by the LIR technique. Furthermore, the performance of the sample as a temperature sensor was studied by obtaining its relative sensitivity and temperature uncertainty. The relative sensitivity presents, as far as can be ascertained, the highest sensitivities recorded to date for organic–inorganic hybrid materials. The temperature uncertainty in the study presented values below 0.15 °C for the whole temperature range. All of these results position [Eu_0.0135_Gd_0.9865_(pta)_3_me-phen] as one of the top candidates for optical temperature sensing in the physiological range.

## Figures and Tables

**Table 1:** Crystal data and structure refinement details.

Compound	[Gd(pta)_3_me-phen] ^a^
CCDC Number	2162029
Empirical formula	C_43_H_27_F_9_GdN_2_O_6_
*M*/gmol^−1^	995.91
Temperature (K)	293
λ/Å	1.54184
Crystal system	Monoclinic
space group	*P*2_1_/*c*
*a, (*Å)	10.0369 (3)
*b,(*Å)	37.5293 (15)
*c,(*Å)	11.1039 (3)
β (°)	91.093 (3)
*V* (Å^3^)	4181.8 (2)
*Z*	4
*D*_calc_/gcm^−3^	1.582
μ/(mm^−1^)	11.05
Theta range/°	4.6–72.3
*R*_int_	0.038
*R*_1_[*I*> 2*σ*(*I*) ^b^	0.0895
w*R*_2_[I > 2*σ*(*I*)] ^c^	0.1575
GOF on*F* ^2 d^	1.053

^a^ The compound is formulated this way for simplicity**.** ^b^ R_1_ = [∑(‖F_o_| − |F_c_‖)/∑|F_o_|]. ^c^ wR_2_ = [∑[w(F_o_^2^ − F_c_^2^)^2^]/∑[w(F_o_^2^)^2^]]^1/2^. ^d^ Goodness-of-fit S = [Σ [w(F_o_^2^–F_c_^2^)^2^]**/**(n–p)]^1/2^.

**Table 2:** Selected bond distances (Å) and angles (º).

Gd1—O2A	2.3361(1)	Gd1—O2C	2.3370(1)
Gd1—O1A	2.3444(1)	Gd1—N2	2.5491(1)
Gd1—O1C	2.3470(1)	Gd1—N1	2.5880(1)
Gd1—O2B	2.3574(1)		
O2A—Gd1—N2	85.648(1)	O1C—Gd1—O2C	71.629(1)
O1A—Gd1—O2A	71.780(1)	O2B—Gd1—O2C	76.567(1)
O1A—Gd1—O1C	85.241(1)	N1—Gd1—O2B	70.877(1)
N1—Gd1—N2	62.421(1)		

**Table 3:** Intramolecular hydrogen bonding distances in Å.

F3A-H16A	2.3378(1)	O2A—H19A	2.4159(1)
F3C-H16C	2.3769(1)	O2B—H23B	2.4937(1)
F1B-H16B	2.3629(1)	O2C—H19C	2.4520(1)

**Table 4:** List of optical temperature sensors based on Eu^3+^-doped lanthanide ions, ranked by relative sensitivity.

Host	Doped Ions	Temp Range (K)	T_max_ (K)	Max S_r_ (% K^−1^)	Refs
H_4_L	Tb^3+^/Eu^3+^	4–290	4	31	[31]
BTC	Tb^3+^/Eu^3+^	298–383	383	16	[32]
(L^1^)(HL^1^)	Eu^3+^	80–180	125	12	[33]
(pta)_3_me-phen	Eu^3+^/Gd^3+^	296–335	335	11.4	This work
CaMoO_4_	Tb^3+^/Eu^3+^	298–603	603	9.50	[34]
HOF-TCBP	Eu^3+^	297–377	297	5.79	[35]
L(DMF)_2_(NO_3_)	Tb^3+^/Eu^3+^	10–300	250	4.90	[36]
UiO-66	Zr^3+^/Eu^3+^	237–337	337	4.67	[37]
Ln@Al(OH)(bpydc)	Tb^3+^/Eu^3+^	283–333	333	3.00	[38]
ZJU88 ⊃perylene	Eu^3+^	293–353	293	1.28	[11]
β-NaY_0.8_Gd_0.2_F_4_	Tb^3+^/Eu^3+^	303–563	303	0.76	[39]
POM@MOF	Tb^3+^/Eu^3+^	60–360	60	0.71	[40]
CGS	Tb^3+^/Eu^3+^	313–473	473	0.56	[41]
Ca_8_ZnLa(PO_4_)_7_	Tb^3+^/Eu^3+^	298–498	298	0.53	[42]
[Ln(hfa)_3_(dpbp)]n	Tb^3+^/Eu^3+^	200–300	200	0.52	[43]
Gd_2_(MoO_4_)_3_	Tb^3+^, Eu^3+^	80–450	270	0.50	[44]
NaYF_4_	Ce^3+^/Tb^3+^/Eu^3+^	303–573	573	0.46	[45]
CaF_2_	Tb^3+^/Eu^3+^	21–320	21	0.40	[46]
YF_3_	Tb^3+^/Eu^3+^	303–563	563	0.38	[47]
Borate glass	Tb^3+^/Eu^3+^	353–573	573	0.35	[48]
[Ln(bdc)_1.5_(H_2_O)_2_]	Tb^3+^/Eu^3+^	290–320	318	0.31	[49]
SiO_2_–Y_2_O_3_	Tb^3+^/Eu^3+^	298–333	303	0.29	[50]
YF_3_	Ce^3+^/Tb^3+^/Eu^3+^	303–563	563	0.20	[47]
Sr_3_GdNa(PO_4_)_3_F	Tb^3+^/Eu^3+^	303–483	303	0.16	[51]
YF_3_ glass	Tb^3+^/Eu^3+^	303–563	563	0.13	[52]
[Ln_2_(D-cam)(Himdc)_2_(H_2_O)_2_]	Tb^3+^/Eu^3+^	100–450	450	0.11	[53]

## Data Availability

The data presented in this study are available on request from the corresponding author. The data are not publicly available due to own empirical research of corresponding author.
